# Examining the Relationship between Exercise Dependence, Disordered Eating, and Low Energy Availability

**DOI:** 10.3390/nu13082601

**Published:** 2021-07-28

**Authors:** Megan A. Kuikman, Margo Mountjoy, Jamie F. Burr

**Affiliations:** 1 The Human Performance and Health Research Laboratory, Department of Human Health and Nutritional Sciences, University of Guelph, Guelph, ON N1G 2W1, Canada; mkuikman@uoguelph.ca; 2 Department of Family Medicine, Michael G. DeGroote School of Medicine, McMaster University, Hamilton, ON L8P 1H6, Canada; mmsportdoc@mcmaster.ca

**Keywords:** exercise addiction, compulsive exercise, relative energy deficiency in sport, LEAF-Q

## Abstract

Both dietary and exercise behaviors need to be considered when examining underlying causes of low energy availability (LEA). The study assessed if exercise dependence is independently related to the risk of LEA with consideration of disordered eating and athlete calibre. Via survey response, female (*n* = 642) and male (*n* = 257) athletes were categorized by risk of: disordered eating, exercise dependence, disordered eating and exercise dependence, or if not presenting with disordered eating or exercise dependence as controls. Compared to female controls, the likelihood of being at risk of LEA was 2.5 times for female athletes with disordered eating and >5.5 times with combined disordered eating and exercise dependence. Male athletes with disordered eating, with or without exercise dependence, were more likely to report signs and symptoms compared to male controls-including suppression of morning erections (OR = 3.4; *p* < 0.0001), increased gas and bloating (OR = 4.0–5.2; *p* < 0.002) and were more likely to report a previous bone stress fracture (OR = 2.4; *p* = 0.01) and ≥22 missed training days due to overload injuries (OR = 5.7; *p* = 0.02). For both males and females, in the absence of disordered eating, athletes with exercise dependence were not at an increased risk of LEA or associated health outcomes. Compared to recreational athletes, female and male international caliber and male national calibre athletes were less likely to be classified with disordered eating.

## 1. Introduction

When the energy demands of exercise are unmatched with sufficient energy intake, a state of low energy availability (LEA) can occur [1,2]. Energy availability represents the dietary energy remaining after accounting for exercise training for all other metabolic processes and is operationally defined as energy intake minus exercise energy expenditure normalized to fat free mass [3]. Low energy availability may result in metabolic and endocrine alterations [4,5] and underlies the syndrome of relative energy deficiency in sport (RED-S), which is defined by impaired physiological function with various health and performance consequences [1,2]. A state of LEA may inadvertently occur from an unintentional mismatch of energy supply and demand, or it can also result from disordered eating or a clinically diagnosable eating disorder [1]. While the characteristics of a clinical eating disorder and disordered eating differ, it is the energy restriction component that may lead to a state of LEA and athletes are more likely to present with disordered eating than a clinical eating disorder [6]. As such, for the remainder of this paper, we will use the terminology of disordered eating to encompass both disordered eating and eating disorder behaviours unless discussing literature that has involved a clinical diagnosis.** Notably, while disordered eating is less prevalent in male compared to female athletes [7,8,9,10], disordered eating in male athletes may be underreported due to stigmatization [11] and few studies have examined the relationship between disordered eating and LEA in male athletes.

As energy availability is determined by both energy intake and exercise energy expenditure, both dietary and exercise behaviours, including problematic exercise behaviours such as exercise dependence, need be considered when examining underlying causes of LEA. Exercise dependence, also known as exercise addiction, is conceptualized as being akin to substance dependence disorders such that exercise is seen as an addictive behaviour that is intrinsically motivated through an influence on positive affect [12]. However, exercise dependence is not formally recognized within the Diagnostic and Statistical Manual for Mental Disorders (DSM-V) [13]. Exercise dependence can occur in conjunction with or as a result of disordered eating, which is known as secondary exercise dependence [12]. However, primary exercise dependence, or that which occurs in the absence of disordered eating is also possible [14,15,16,17]. In situations of primary exercise dependence, continual exercise is undertaken solely for the psychological gratification resulting from the exercise behaviour rather than being the result of another pathology [12].

While disordered eating has long been recognized to lead to the development of LEA [1,2], the role of exercise dependence independent of disordered eating in the development of LEA has yet to be examined. Preliminary studies in athletes with symptoms of exercise dependence report elevations in some biochemical markers indicative of LEA [18,19]. However, these findings are limited, and a causative role of primary vs. secondary exercise dependence has not been elucidated. LEA in situations of secondary, but not primary, exercise dependence could contribute to the disordered eating behaviours rather than the exercise dependence per se. Using validated screening tools, the primary objective of this study was to investigate if primary exercise dependence increases the risk of LEA in female athletes. Given that a higher risk of LEA [20,21], exercise dependence [22,23,24], and disordered eating [25,26] has been reported in athletes competing at a higher athlete caliber, we also aimed to compare results across athletes of different levels of competition. While similar tools are not currently available for male athletes, a secondary aim was to screen for symptoms of LEA in male athletes to address the lack of studies examining the potential relationship between disordered eating and LEA. We hypothesized that athletes at risk of both primary and secondary exercise dependence would be at an increased risk of LEA, as would athletes competing at the highest level of competition.

## 2. Materials and Methods

### 2.1. Participants

Athletes were invited to complete an anonymous electronic questionnaire that was e-mailed to team leads at national sports organizations and shared on social media platforms between June 2020–April 2021. Athletes ≥18 years of age, from any country, who were training for and competing at any level of sport, were eligible. This study was approved by the University of Guelph Research Ethics Board (REB# 19-10-007).

### 2.2. Questionnaire

Table 1 summarizes the questionnaires that were included in the online survey. Based on the Exercise Dependence Scale and Eating Disorder Examination Questionnaire scoring systems (see Table 1), athletes were classified into one of the following categories: (1) Disordered eating: at risk of disordered eating only; (2) Primary exercise dependence: at risk of exercise dependence only; (3) Secondary exercise dependence: at risk of both disordered eating + exercise dependence; (4) Control athletes: not at risk of disordered eating or exercise dependence.

To assess the impact of disordered eating and exercise dependence status on LEA risk, females completed the Low Energy Availability in Females Questionnaire (LEAF-Q) [27]. The LEAF-Q was validated within athletic populations and implemented using the scoring system (see Table 1) [27]. As no validated survey exists for male athletes, to explore symptoms of LEA in males with disordered eating and exercise dependence status, male athletes were asked questions to assess for health outcomes across the variables included within the LEAF-Q: injury history, gastrointestinal symptoms, and reproductive dysfunction [27]. In place of menstrual dysfunction, male athletes were asked questions about erectile dysfunction. Both male and female athletes were further questioned about history of bone stress fractures given the increased risk reported in athletes with LEA [28], as well as additional questions in regards to sport discipline, training program, anthropometrics, and highest level of competition.

### 2.3. Statistical Analysis

Normality of data was assessed using the skewness and kurtosis of the distribution. One-way ANOVA was used to compare baseline participant characteristics, stress fracture history, Eating Disorder Examination Questionnaire results, and Exercise Dependence Scale results between athletes with secondary exercise dependence, disordered eating, primary exercise dependence and control athletes, with Tukey’s post-hoc analysis, as required. For non-normally distributed variables, a Kruskal–Wallis test was used with a Bonferroni adjusted post-hoc analysis. Body mass index, weekly strength exercise, and weekly mobility exercise were analyzed using non-parametric statistics as well as years competing and global Eating Disorder Examination Questionnaire score in males. Multinomial regression was used to yield exponentiating regression coefficients when assessing risk of being classified as at risk of LEA, menstrual dysfunction, history of >1 bone stress fracture as well as risk of being classified as disordered eating, exercise dependence and/or at risk of LEA with competitive status. When survey questions were ordinal in nature (missed training days due to injuries, gastrointestinal symptoms, and erectile dysfunction), an ordinal regression was used to yield an exponentiating regression coefficient. For both multinomial and ordinal regression, when assessing disordered eating and exercise dependence status, the control athletes served as the reference group and for differences across athlete calibre, recreational athletes were used as the reference group. Analyses were completed using Statistical Package for the Social Sciences (SPSS, V.27, IBM, Chicago, IL, USA), with an a priori alpha of <0.05 for significance.

## 3. Results

The electronic survey was completed by 650 female and 276 male athletes. A small proportion of survey responses were excluded for incomplete data (eight female/19 male). The self-reported distribution of female/male athlete respondents by sport classification was: 84% (*n* = 565/188) endurance sport athletes such as cycling, long-distance running; 8% (*n* = 32/38) mixed sport athletes such as soccer, rugby; 5% (*n* = 30/16) power sport athletes such as sprinting, shot-putting; and 1% (*n* = 11/0) skill sport athletes such as archery, equestrian. The remaining 19 athletes (four female/15 male) did not specify their primary sport. The distribution of female/male athlete across self-reported level of competition was: recreational (*n* = 148/62), collegiate (*n* = 217/38), national (*n* = 156/90), and international athletes (*n* = 119/66). Most survey respondents were from North America (*n* = 652), with further participation from Europe (*n* = 152), Australia (*n* = 44), Africa (*n* = 15), Asia (*n* = 3), and South America (*n* = 3). Thirty athletes did not specify their country.

### 3.1. Participant Characteristics

Of the 642 female athletes, 151 (24%) were classified with disordered eating, 82 (13%) with secondary exercise dependence, and 23 (4%) with primary exercise dependence. The remaining 386 (60%) female athletes served as the control athletes. Of the 257 male athletes, 57 (22%) were classified with disordered eating only, 13 (5%) with secondary exercise dependence, and six (2%) with primary exercise dependence. The remaining 181 (70%) male athletes served as the control athletes. Age, body mass index, and training information of participants with disordered eating and exercise dependence status are presented in Table 2.

### 3.2. Eating Disorder Examination Questionnaire and Exercise Dependence Scale Results

As expected, both male and female respondents with primary and secondary exercise dependence had a higher total score on the Exercise Dependence Scale than athletes with disordered eating and control athletes, with athletes with disordered eating recording a higher total score than that of control athletes. Moreover, not surprisingly, athletes with secondary exercise dependence and disordered eating had higher global scores on the Eating Disorder Examination Questionnaire than control athletes. However, female athletes with secondary exercise dependence and disordered eating also had higher global scores than athletes with primary exercise dependence and, interestingly, athletes with secondary exercise dependence scored higher than athletes with disordered eating. Differences and raw data across all Eating Disorder Examination Questionnaires and Exercise Dependence Scale subscales are offered in Supplementary Table S1.

### 3.3. Risk of LEA and Associated Health Outcomes with Exercise Dependence and Disordered Eating Status

#### 3.3.1. Risk of LEA

**Figure 1:**
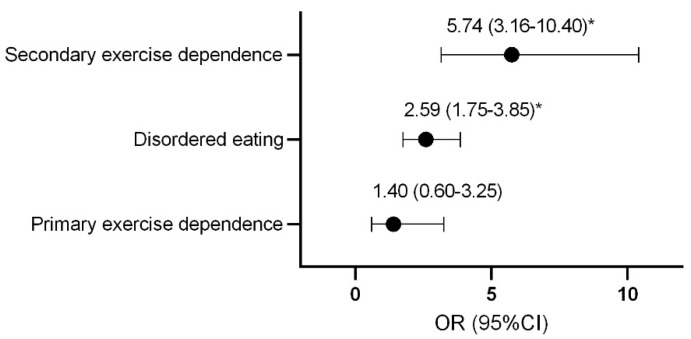
Odds ratio and confidence interval of female athletes being classified as at risk of low energy availability with disordered eating and exercise dependence status. * *p* < 0.0001. OR, odds ratio; CI, confidence interval.

Compared to control athletes, females with secondary exercise dependence or disordered eating were more likely to be classified as at risk of LEA (Figure 1).

#### 3.3.2. Reproductive Symptoms

**Figure 2:**
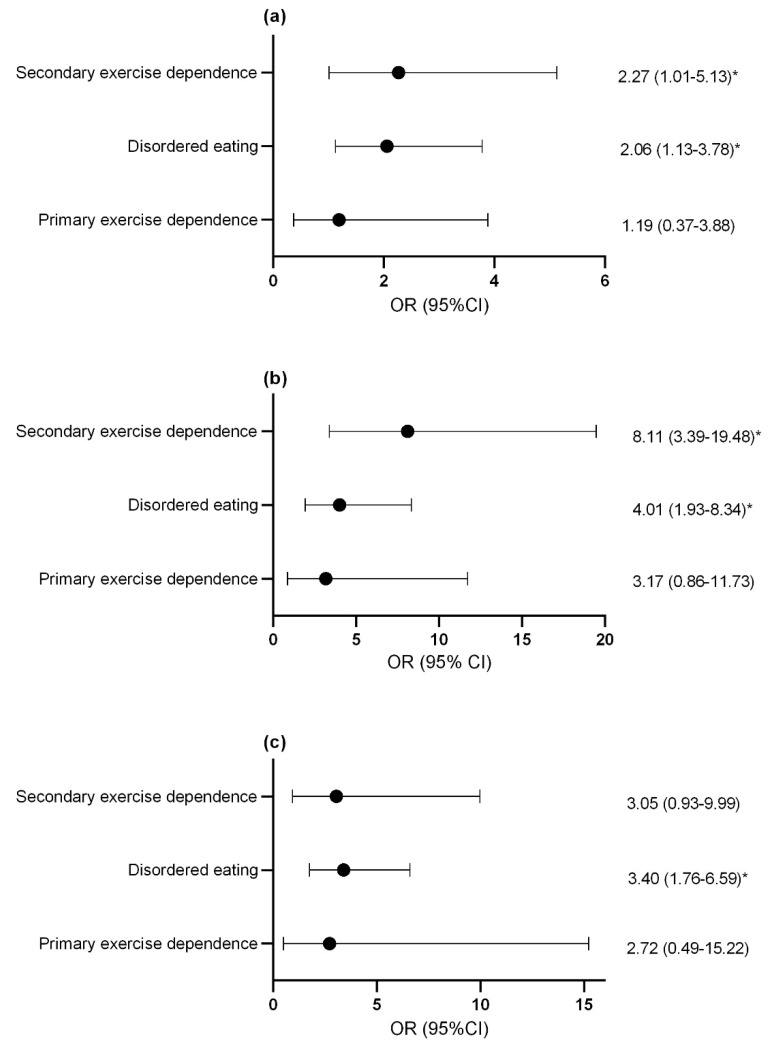
Odds ratio and confidence interval of (**a**) previous menstrual dysfunction (**b**) current menstrual dysfunction and (**c**) suppression of morning erections with disordered eating and exercise dependence status. * *p* < 0.05. OR, odds ratio; CI, confidence interval.

Compared to controls, females with secondary exercise dependence or disordered eating were more likely to report previous menstrual dysfunction (Figure 2a) and current menstrual dysfunction (Figure 2b). Males with disordered eating were more likely to report decreased morning erectile function compared to controls and this trended toward statistical significance in athletes with secondary exercise dependence (*p* = 0.06; Figure 2c).

#### 3.3.3. Gastrointestinal Symptoms

**Figure 3:**
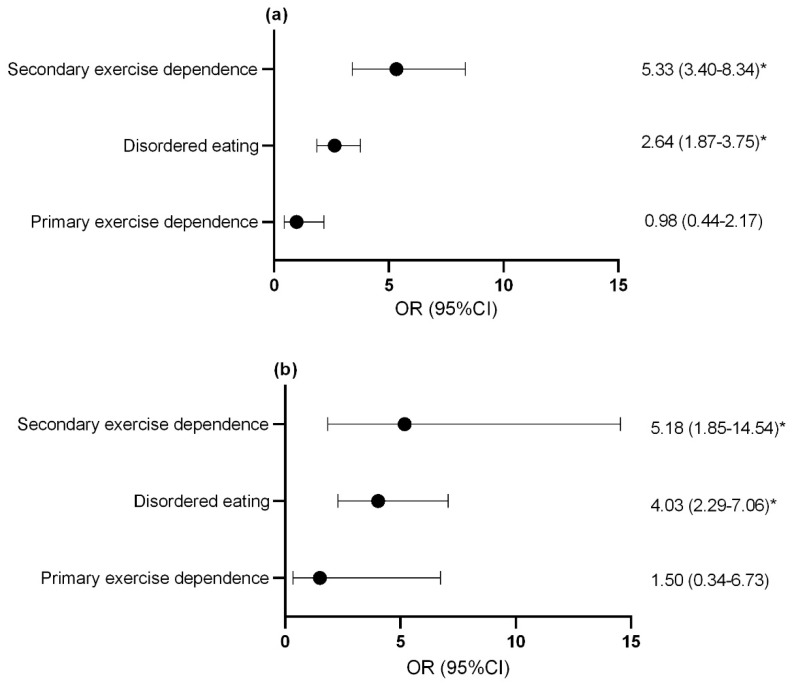
Odds ratio and confidence interval of increased gas and bloating in (**a**) females and (**b**) males with disordered eating and exercise dependence status. * *p* < 0.05. OR, odds ratio; CI, confidence interval.

For both females (Figure 3a) and males (Figure 3b), athletes with secondary exercise dependence and disordered eating athletes were at an increased risk of reporting gastrointestinal symptoms at an increased frequency compared to controls.

#### 3.3.4. Injuries and Bone Stress Fractures

**Figure 4:**
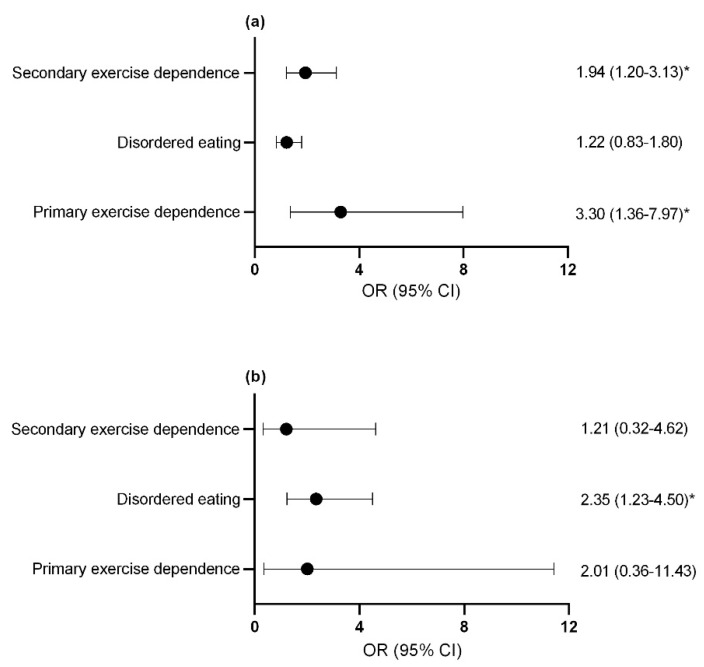
Odds ratio and confidence interval of at least 1 previous bone stress fracture in (**a**) females (**b**) males. * *p* < 0.05. exercise dependence, exercise dependence. OR, odds ratio; CI, confidence interval.

Compared to control athletes, female athletes with primary and secondary exercise dependence (Figure 4a) and male athletes with disordered eating were more likely to report a previous bone stress fracture (Figure 4b). The assumption that the parameters were the same for all categories (test of parallel lines) was violated when considering the risk of missed training days due to injuries for both male and female athletes. As such, a multinomial regression was used in place of ordinal regression to assess the risk of missing ≥22 training days or competition days due to injury over the last year with female athletes with secondary exercise dependence being identified for this increased risk (OR = 2.25; 95% CI = 1.39–3.65; *p* = 0.001). Similarly, male athletes with secondary exercise dependence were more likely to report missing ≥22 training days or competition days due to overload injuries over the last year (OR = 5.73; 95% CI = 1.34–24.54; *p* = 0.019). No significant difference was seen for missed training days due to acute injuries in male athletes with disordered eating and exercise dependence status.

### 3.4. Risk of Disordered Eating, Exercise Dependence and LEA with Athlete Calibre

Three athletes (2F/1M) did not include competitive status and were excluded from this analysis. While no difference was seen across athlete calibre for risk of LEA and exercise dependence, compared to female recreational athletes, international calibre female athletes were 46% less likely to be classified with disordered eating (OR = 0.54; 95% CI = 0.30–0.99; *p* = 0.046). A similar trend was seen in male athletes such that national athletes were 54% less likely (OR = 0.46; 95% CI = 0.22–0.98; *p* = 0.045) and international athletes 66% less likely (OR = 0.34; 95% CI = 0.14–0.78; *p* = 0.012) to be classified with disordered eating compared to recreational athletes.

## 4. Discussion

The aim of this study was to assess if athletes with exercise dependence, both with and without disordered eating, were at an increased risk of LEA and differences across the spectrum of athlete calibre. The major novel finding of this study was that while exercise dependence did increase the risk of LEA and associated health outcomes, this was only noted when exercise dependence co-occurred with disordered eating. We must also reject our hypothesis regarding the risk of LEA, disordered eating, and exercise dependence, which were not increased among athletes competing at the highest level. Rather, recreational athletes were more likely to be classified with disordered eating for both males and females compared to international caliber athletes, and at relatively similar rates for the responses received.

Disordered eating behaviors and diagnosed eating disorders are recognized as causes of LEA in athletes [6]. As such, it is not surprising that compared to control athletes, male and female athletes classified as at risk of disordered eating were at an increased risk of LEA and associated health outcomes. However, for females with secondary exercise dependence there was an exacerbated risk of LEA compared to athletes with disordered eating in isolation. This may be due to the higher psychopathology in athletes with secondary exercise dependence given they had a higher Eating Disorder Examination Questionnaire global score as well as also reporting engaging in significantly more aerobic exercise than athletes with disordered eating. To our knowledge, no study has examined differences in disordered eating psychopathology between athletes with secondary exercise dependence and those with disordered eating alone. However, in those with a clinical eating disorder, compulsive exercise is associated with greater eating disorder pathology [33,34]. Compulsive exercise represents an urge to perform exercise with the intent to escape anxiety that arises from the imagined negative consequences of not exercising [12] and better reflects exercise behaviours that are secondary to disordered eating than exercise dependence [12,35,36]. As such, the problematic exercise behaviours that led to an exacerbated risk of LEA in athletes with secondary exercise dependence may be more reflective of compulsive exercise than exercise dependence.

While male athletes with disordered eating, both with and without co-occurring exercise dependence, reported a greater risk of health outcomes within the RED-S model, unlike female athletes, an exacerbated risk was not noted in athletes with secondary exercise dependence. While this may suggest sex-based differences in regard to the effect of secondary exercise dependence, these differences may be due to the questions used to assess health outcomes associated with LEA given these are not yet validated. Regardless, our results reinforce that disordered eating is also a concern among male athletes [37], and extends this finding by demonstrating that disordered eating both with or without exercise dependence may increase the risk of various health outcomes, and provides insight into possible warning signs of disordered eating in male athletes. Evidently, disordered eating and LEA are not just a female athlete problem, and male athletes can also suffer negative health consequences due to disordered eating behaviours.

A novel finding of this study was that primary exercise dependence did not increase the reported risk of LEA or associated health outcomes. This is contrary to preliminary evidence on which our hypothesis was based suggesting that exercise dependence may lead to LEA in males that scored low on disordered eating measures [18,19]. These conflicting findings may be due to the surrogate markers of LEA measured in these studies [18,19] that can be influenced by factors beyond LEA, such as the impact of exercise on testosterone and cortisol levels [38]. We found that athletes with primary exercise dependence did not differ from control athletes on many measures, including weekly exercise, and for male athletes the global measures of eating disorder were not different between these two groups. On the other hand, female athletes with primary exercise dependence did have a higher Eating Disorder Examination Questionnaire global score than control athletes. While measures were still below the threshold indicative of disordered eating psychopathology, it is possible that athletes were falsely classified with primary exercise dependence instead of secondary exercise dependence, as has been reported by others [39]. Notably, female athletes with primary exercise dependence were at an increased risk of a previous bone stress fracture. As an increased risk of bone stress fracture has been reported in athletes with LEA [28]—but athletes with primary exercise dependence were not at an increased risk of LEA—this higher incidence of injury could be due to the variety of other factors contributing to stress fractures, such as biomechanical and environmental factors [40]. Alternatively, as the specific timing of previous fractures was not assessed, it is possible that these occurred at a time when athletes were in a state of LEA.

The low prevalence of primary exercise dependence (~2–4%) suggests that overt exercise dependence without co-occurring disordered eating is relatively uncommon. Notably, there is debate as to whether primary exercise dependence is a health concern warranting separate diagnosis [39,41]. While our results do not support an increased risk of LEA in those with primary exercise dependence, excessive exercise could interfere with other areas of life through time conflicts [42] and potentially lead to overreaching or overtraining syndrome [43]. However, in our population, athletes with primary exercise dependence did not report engaging in more exercise than control athletes. Further research is needed to assess the role of primary exercise dependence in athlete health and wellbeing.

Despite no differences in the risk of LEA in females or exercise dependence across athlete calibre, we did observe the risk of disordered eating in both males and females to be lowest amongst those competing at the highest level of competition. Existing data on the relationship between rates of disordered eating across athlete calibre have reported a higher risk amongst athletes of higher calibre [25,26], but this is not a universal finding [44,45,46]. These conflicting findings may reflect different caliber of athletes being included in the “higher” versus “lower” competitive group or the type of athletes included in these studies. In our study, the lower risk of disordered eating amongst international athletes and male national athletes compared to their recreational counterparts may represent a selection factor, such that the health and performance implications of improper fueling could preclude success to progress to the international level. A higher drive for thinness is associated with an increased incidence of musculoskeletal injuries in female athletes [47], and disordered eating related injury could certainly interfere with athletic success due to loss of training time [48]. Differences across athlete calibre may also relate to underlying motivations for training and competing, as initiating training to lose weight is associated with an increased risk of disordered eating development [49]. It is likely that, compared to athletes competing on an international stage, lower calibre athletes have more autonomy to affect decisions about participation and training load in this regard, and less access to support systems including dietitians, targeted professional coaching, and clinical support teams. Future research is needed to assess underlying factors that may have contributed to the increased risk of disordered eating seen in recreational athletes as this may be a prevalent but unaddressed concern that warrants preventative strategies.

As with all research, the current study is not free from limitations, including the fact that this investigation was cross-sectional in nature, precluding insight into cause and effect. While validated screening tools were sought, the LEAF-Q has only been validated in endurance athletes and no such validated tool is available for male athletes-an area worthy of future attention and expansion. Additionally, both the Eating Disorder Examination Questionnaire and Exercise Dependence Scale are screening tools and not diagnostic. The data provided was self-reported in nature and dependent on an athlete’s honesty and understanding of the questions asked. The survey was available only in English, which likely led to the majority of responses being from North American athletes and thus, limits applicability to under-represented populations. It is possible that self-selection bias, such that those that have an interest in disordered eating and RED-S may have been more likely to participate, could impact these results to an unknown extent [50]. Furthermore, given the methods used to promote the survey, we were unable to determine our response rate. Responses may also change across a competitive season, such as leading up to a major competition, and exercise dependence in some competitive athletes may have been overshadowed by “normal” training practices in the sport, which nullify decisions around exercise behaviours that more recreational athletes might face. Notably, the electronic survey was circulated during a period when most competitions were unexpectedly cancelled due to the COVID-19 pandemic.

## 5. Conclusions

Given the low prevalence of primary exercise dependence, athletes exhibiting problematic exercise behaviours should also be screened for disordered eating. In situations of problematic exercise contributing to the development of RED-S, clinical assessment and treatment must not only address dietary patterns, but also exercise behaviours. Athletes with primary exercise dependence were not at increased risk of LEA and associated health outcomes, but further research is needed to determine if primary exercise dependence is a concern relevant to athlete health that warrants a separate diagnosis. Like female athletes, male athletes with disordered eating can demonstrate concerning health consequences within the RED-S model. More research is needed to examine the relationship between disordered eating and LEA in male athletes and the association between exercise dependence and LEA in other types of sports given the majority of athletes in this study competed in endurance sports.

## Figures and Tables

**Table 1:** Summary of questionnaires used to classify athletes as at risk of exercise dependence, disordered eating, risk of low energy availability, and associated health outcomes.

Outcome	Questionnaire	Measures
Exercise dependence	Exercise Dependence Scale [29]	21-items rated on a 6-point Likert scale to compute 7 subscales (3 items/subscale): tolerance, withdrawal, intention effect, lack of control, time, reduction in other activities, and continuance Total score computed by summing subscales For analysis, athletes were classified as at-risk of exercise dependence if score > 15 on >3 subscales
Disordered eating	Eating Disorder Examination Questionnaire [30]	22-items rated on a 6-point Likert scale to compute 4 subscales: restraint, eating concern, shape concern, and weight concern Mean global score computed from subscales For analysis, athletes classified as at risk of disordered eating if global score ≥ 2.5 in females [31] and ≥1.68 in males [32]
LEA in females	Low Energy Availability in Females Questionnaire [27]	25-questions in regard to injury history, gastrointestinal, and reproductive function For analysis, a score ≥ 8 classified an athlete as at risk of LEA
LEA in males	Unvalidated questionnaire	4-questions assessing acute and overload injury history and severity of gastrointestinal and reproductive symptoms

LEA, low energy availability.

**Table 2:** Age, body mass index, and training information with disordered eating and exercise dependence classification.

	Secondary Exercise Dependence	Disordered Eating	Primary Exercise Dependence	Control	*p*-Value
Female athletes					
Age (year)	26.0 ± 6.5	27.4 ± 8.8	27.7 ± 9.3	28.4 ± 9.0	0.15
BMI (kg/m^2^)	20.1 (18.9–21.5)	20.9 (19.8–22.9) ^‡^	19.7 (18.2–20.8)	20.4 (19.2–21.8)	<0.0001
Years competing	12.0 ± 6.4	11.5 ± 7.4	13.0 ± 9.3	13.0 ± 7.6	0.19
Aerobic (h/week)	11.9 ± 4.6 *	9.6 ± 4.0	11.2 ± 3.6	9.2 ± 3.9	<0.0001
Strength (h/week)	3 (2–5) ^§^	3 (2–4)	3 (2–4)	2 (1–3)	<0.0001
Mobility (h/week)	2 (1–4)	2 (1–3)	1 (1–2)	2 (1–3)	0.21
Male athletes					
Age (year)	29.8 ± 10.1	33.7 ± 10.6	27.4 ± 6.8	34.3 ± 12.4	0.62
BMI (kg/m^2^)	22.9 (20.6–24.1)	23.4 (21.8–25.8) ^§^	22.2 (21.7–25.5)	22.4 (21.0–24.1)	0.03
Years competing	10.0 (5.5–15.50)	10.0 (6.0–19.0)	9.0 (5.0–19.0)	11.5 (8.0–21.0)	0.49
Aerobic (h/week)	11.9 ± 4.1	11.0 ± 4.5	12.4 ± 4.4	10.2 ± 4.5	0.28
Strength (h/week)	2 (1–6)	3 (1–4)	1 (0–4)	2 (1–3)	0.21
Mobility (h/week)	1 (1–2)	2 (1–4)	2 (0–3)	2 (1–3)	0.18

Normally distributed data are shown as mean ± SD, and non-normally distributed data as median and IQR (IQ 25 and IQ 75 percentiles). ^‡^ *p* < 0.05 vs. primary exercise dependence, secondary exercise dependence and control athletes; * *p* < 0.05 vs. disordered eating and control athletes; ^§^ *p* < 0.05 vs. control athletes. BMI, body mass index.

## Data Availability

The data presented in this study are available upon reasonable request from the corresponding author.

## Supplementary Materials

The following are available online at https://www.mdpi.com/article/10.3390/nu13082601/s1, Table S1: Global Eating Disorder Examination Questionnaire and Exercise Dependence Scale results.
