# Supplementary material for: Examining the Relationship between Exercise Dependence, Disordered Eating, and Low Energy Availability
Source: Nutrients. 2021 Jul 28;13(8):2601. doi: 10.3390/nu13082601 (PMC8398044; doi:10.3390/nu13082601)
Supplement: Supplementary file 1 [file nutrients-13-02601-s001.zip › nutrients-1289840-supplementary.pdf]

Supplementary Table S1: Global Eating Disorder Examination Questionnaire and Exercise Dependence Scale results

	Secondary exercise dependence	Disordered eating	Primary exercise dependence	Control	Secondary vs Disordered eating	Secondary vs Primary	Secondary vs Control	Disordered eating vs Primary	Disordered eating vs Control	Primary vs Control
Global Eating Disorder Examination Questionnaire results										
Females										
Eating concern	3.59±1.36	2.54±1.20	1.17±0.80	0.57±0.62	<0.0001	<0.0001	<0.0001	<0.0001	<0.0001	0.01
Shape concern	4.81±0.90	4.34±0.79	2.42±0.86	1.65±1.07	<0.0001	<0.0001	<0.0001	<0.0001	<0.0001	<0.0001
Weight concern	4.42±1.08	3.97±0.90	1.73±0.94	1.35±0.93	<0.0001	<0.0001	<0.0001	<0.0001	<0.0001	0.22
Restraint	4.06±1.37	3.14±1.29	1.57±1.57	0.85±0.87	<0.0001	<0.0001	<0.0001	<0.0001	<0.0001	0.01
Global score	4.22±0.90	3.50±0.74	1.72±0.55	1.10±0.68	<0.0001	<0.0001	<0.0001	<0.0001	<0.0001	<0.0001
Males										
Eating concern	2.20(1.2-4.6)	1.60(1.0-2.3)	0.10(0.0-0.85)	0.20(0.0-0.40)	1.00	0.003	<0.0001	0.004	<0.0001	1.00
Shape concern	3.78±1.18	3.56±1.12	1.31±0.96	0.91±0.69	0.82	<0.0001	<0.0001	<0.0001	<0.0001	0.66
Weight concern	3.25±1.41	2.89±1.08	1.28±0.71	0.68±0.64	0.47	<0.0001	<0.0001	<0.0001	<0.0001	0.28
Restraint	3.12±1.42	2.78±1.45	1.77±1.49	0.77±0.95	0.74	0.07	<0.0001	0.16	<0.0001	0.14
Global score	2.85(2.32-4.39)	2.42(2.10-3.20)	1.23(0.78-1.54)	0.61(0.28-1.01)	1.00	0.15	<0.0001	0.14	<0.0001	0.42
Exercise Dependence Scale results										
Females										
Intention effect	13.45±3.45	8.97±2.87	11.43±4.19	6.71±2.83	<0.0001	0.02	<0.0001	<0.0001	<0.0001	<0.0001
Withdrawal	15.35±1.70	12.77±2.68	14.26±3.21	10.93±3.45	<0.0001	0.44	<0.0001	0.14	<0.0001	<0.0001
Continuance	14.46±3.12	10.22±3.35	12.09±4.67	7.70±3.30	<0.0001	0.01	<0.0001	0.06	<0.0001	<0.0001
Tolerance	15.67±2.28	12.11±3.18	15.57±2.79	10.34±3.61	<0.0001	1.00	<0.0001	<0.0001	<0.0001	<0.0001
Lack of control	14.33±2.90	9.32±3.16	13.39±3.03	6.78±3.13	<0.0001	0.58	<0.0001	<0.0001	<0.0001	<0.0001
Reduction	13.12±2.78	9.46±2.58	11.04±3.43	7.18±2.43	<0.0001	<0.0001	<0.0001	0.03	<0.0001	<0.0001
Time	16.04±1.46	11.97±2.97	16.26±1.79	10.52±3.23	<0.0001	0.99	<0.0001	<0.0001	<0.0001	<0.0001

Supplementary Table S1: Global Eating Disorder Examination Questionnaire and Exercise Dependence Scale results

	Secondary exercise dependence	Disordered eating	Primary exercise dependence	Control	Secondary vs Disordered eating	Secondary vs Primary	Secondary vs Control	Disordered eating vs Primary	Disordered eating vs Control	Primary vs Control
Total score	102.43±10.64	74.81±13.65	94.04±8.92	60.16±14.64	<0.0001	0.05	<0.0001	<0.0001	<0.0001	<0.0001
Males										
Intention effect	12.54±2.90	7.84±2.72	10.50±3.08	6.88±2.92	<0.0001	0.48	<0.0001	0.14	0.13	0.01
Withdrawal	14.85±1.35	11.09±3.78	13.83±4.92	9.24±3.50	<0.0001	0.94	<0.0001	0.27	<0.0001	0.01
Continuance	12.38±4.21	8.86±3.79	12.50±3.83	6.93±3.05	<0.0001	1.00	<0.0001	0.05	<0.0001	<0.0001
Tolerance	14.69±2.87	11.42±3.23	15.83±1.33	10.67±3.10	<0.0001	0.88	<0.0001	0.01	0.38	<0.0001
Lack of control	11.69±3.55	8.61±3.47	9.67±3.98	6.61±2.78	0.01	0.52	<0.0001	0.85	<0.0001	0.07
Reduction	13.46±2.79	9.07± 2.57	11.50±2.59	7.54±2.73	<0.0001	0.45	<0.0001	0.15	<0.0001	<0.0001
Time	15.92±1.71	12.04±3.50	14.67±4.50	10.69± 3.12	<0.0001	0.86	<0.0001	0.22	0.12	0.03
Total score	95.54±9.21	68.93±14.15	88.50±8.87	58.83±12.78	<0.0001	0.69	<0.0001	<0.0001	<0.0001	<0.0001

Normally distributed data are shown as mean and SD, and non-normally distributed data as median and IQR (IQ 25 and IQ 75 percentiles).
